# Direct Imaging of the Crystalline Domains and Their Orientation in the PS-*b*-PEO Block Copolymer with 4D-STEM

**DOI:** 10.1021/acs.macromol.3c02231

**Published:** 2024-04-18

**Authors:** Min Chen, Karen C. Bustillo, Vivaan Patel, Benjamin H. Savitzky, Hadas Sternlicht, Jacqueline A. Maslyn, Whitney S. Loo, Jim Ciston, Colin Ophus, Xi Jiang, Nitash P. Balsara, Andrew M. Minor

**Affiliations:** † Department of Materials Science and Engineering, University of California, Berkeley, California 94720, United States; ‡ Materials Science Division, Lawrence Berkeley National Laboratory, Berkeley, California 94720, United States; § National Center for Electron Microscopy, Molecular Foundry, Lawrence Berkeley National Laboratory, Berkeley, California 94720, United States; ∥ Department of Chemical and Biomolecular Engineering, University of California, Berkeley, California 94720, United States; ⊥ Department of Chemical and Biological Engineering, University of Wisconsin–Madison, Madison, WI 53706, United States

## Abstract

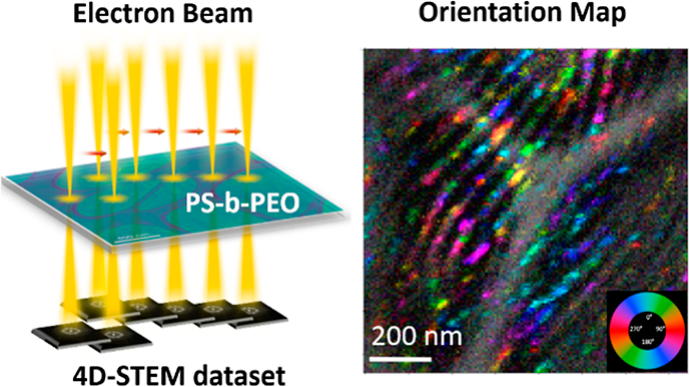

The arrangement of crystalline domains in semicrystalline polymers is key to understanding how to optimize the nanostructured morphology for enabling better properties. For example, in polystyrene-*b*-poly(ethylene oxide) (PS-*b*-PEO), the degree of crystallinity and arrangement of the crystallites within the PEO phase plays a crucial role in determining the physical properties of the electrolyte. Here, we used four-dimensional scanning transmission electron microscopy to directly visualize the crystal domains within the PEO-rich region of the PS-*b*-PEO block copolymer and show the relative angle of the domain with respect to the PEO–PS interface. As demonstrated here, our analysis method is applicable to other electron-beam sensitive materials, especially semicrystalline polymers, to unveil their local phase condition and distribution.

## Introduction

**Figure 1:**
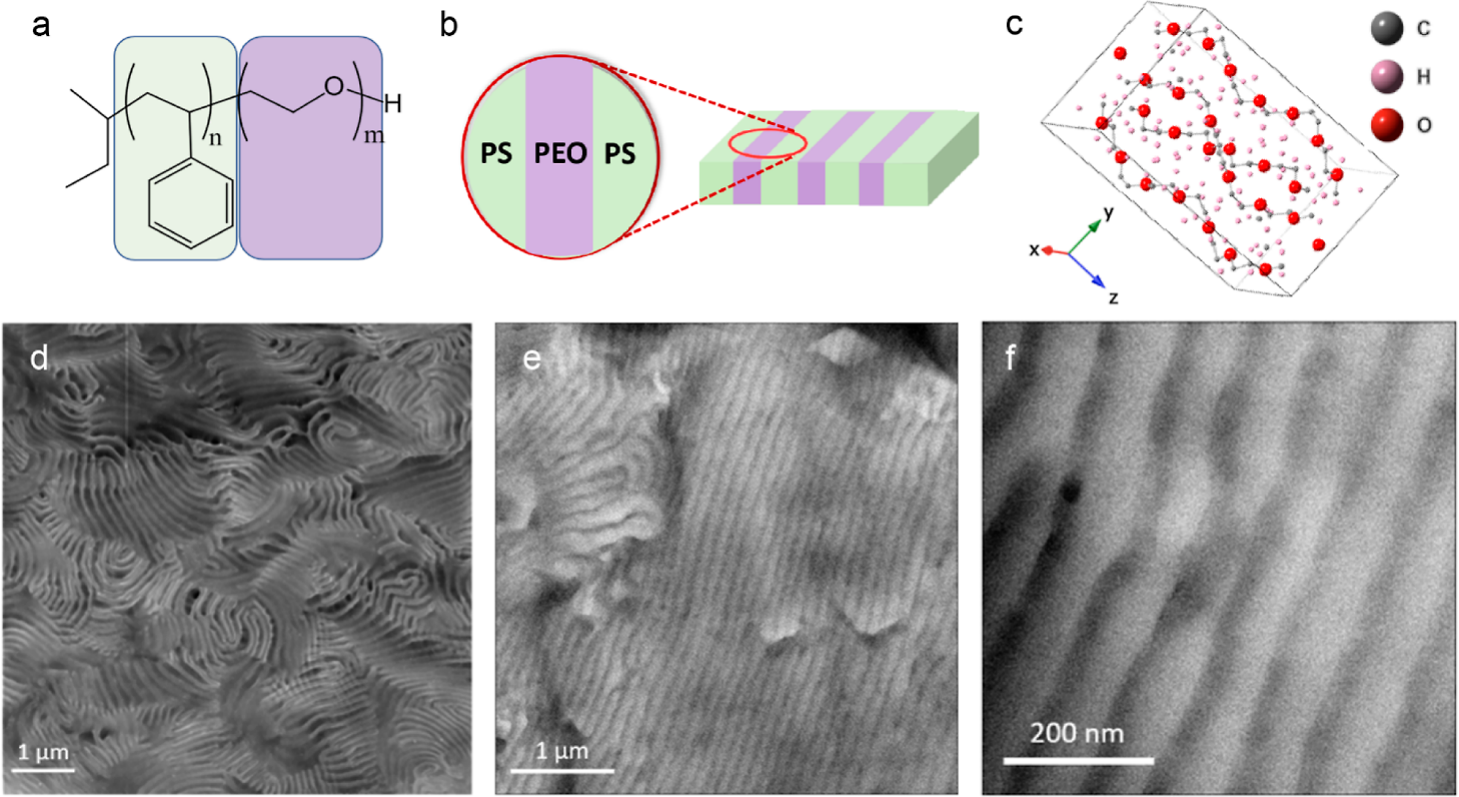
Morphology of PS-*b*-PEO: (a) chemical structure of PS-*b*-PEO (200–222), PS is shown in green, and PEO shown in purple; (b) schematic of the lamellar morphology; (c) unit cell of pure PEO; (d–f) RuO_4_-stained TEM images showing the 40–60 nm block widths; (d) HAADF-STEM image with PEO being bright; (e,f) BF with PEO shown dark.

The confinement of crystalline domains within microphases formed by block copolymers is a subject of long-standing interest. Polymer chains form chain-folded crystalline domains, which coexist with amorphous regions.^1^ For example, a nanostructured block copolymer electrolyte, polystyrene-*b*-poly(ethylene oxide) (PS-*b*-PEO), has emerged as a promising candidate in lithium metal batteries due to its ability to both transport ions and suppress lithium metal dendrites.^2^ PS-*b*-PEO, sometimes called SEO for styrene-ethylene oxide, separates into alternating PEO-rich and PS-rich domains (Figure 1a,b). The PEO domain dissolves the lithium salt effectively, enabling ion transport, while the PS phase provides the necessary mechanical stiffness. In the lamellar morphology of this electrolyte, the PEO is semicrystalline, while the PS is amorphous. Ion transport is limited to the PEO block amorphous part. Since ion transport may be affected by domain distribution and the degree of crystallinity,^3,4^ understanding the nature of the crystallinity inside the PEO domains is crucial since the salt is preferentially located in these regions.^5,6^ In this paper, we use four-dimensional scanning transmission electron microscopy (4D-STEM) to probe the crystalline domains of the PEO phase to measure the size of the domains and their preference for domain orientation with respect to the long directions of the PEO block.

Numerous methods have been employed in previous studies to investigate the structures of crystalline block copolymers. A significant number of these studies have been conducted on polystyrene-*b*-poly(ethylene oxide) (PS-*b*-PEO). Using the width of the X-ray scattering signal, previous studies suggested that the structure within the PEO domain is polycrystalline and randomly oriented.^7,8^ X-ray scattering, a commonly used technique, provides average structural information with spatial resolution on the order of hundreds of nanometers, much larger than the widths of most block phases. In addition, X-ray studies cannot detect the localized heterogeneous structure at the interfaces. Atomic force microscopy (AFM) has been utilized to map phases in polymers using mechanical contrast and to measure dimensional spacings.^9^ However, AFM is a surface technique and cannot provide crystallographic information. Traditionally, electron microscopy studies of PS-*b*-PEO have utilized heavy element staining (such as that of OsO_4_ and RuO_4_) to increase the image contrast and protect the morphology from electron beam damage. These heavy metal stains function by replacing or being absorbed by the organic material, which improves the image contrast but indisputably changes the sample. Consequently, information obtained from stained samples is restricted to the block nature of PS and PEO, with no insights into the degree of local crystallinity or crystal orientation within the blocks themselves. The spatial distribution of PEO domain orientations and any preferential orientation with respect to the PS–PEO interface is of particular importance.

The crystal structure of pure PEO is monoclinic in most cases. Previous studies report the lattice constants to be *a* = 8.05 Å, *b* = 13.04 Å, *c* = 19.48 Å and an angle between the a and *c* axis of 125.40°.^10,11^ A .cif file (Crystallographic Information File, a standard text file format for representing crystallographic information) was built based on the X-ray scattering data from this reference, and the model for the unit cell is shown in Figure 1c. The conventional representation of a crystalline polymer domain shows folding of the polymer chains to make a cuboid or “brick”. Previous reports have created models that show different orientations of the domains within the block PEO phase, but confirmation of these models in the real space is needed. Not all PEO phases are alike.^12,13^ In addition to the variability in molecular weight, which influences crystallinity, it is reported that the crystallization temperature, mechanical stress, and confinement can be used to change the orientation of the crystalline domains.^14,15^ Being able to control the chain axis direction by creating a desired texture, with either stress or temperature, provides an added control for the ion transport, and direct imaging of the crystallinity could be used to measure these subtle changes.

**Figure 2:**
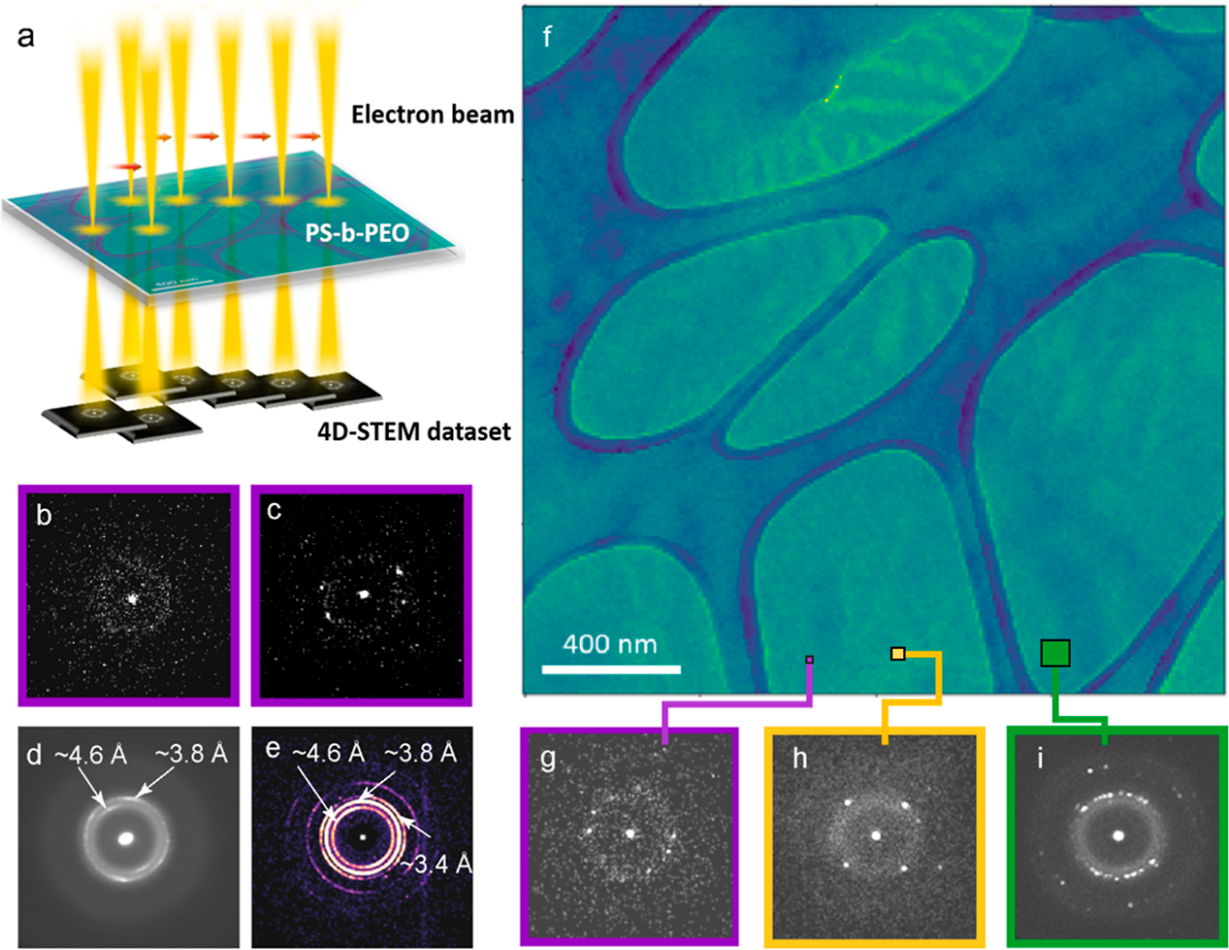
4D-STEM experiment; (a) schematic showing DPs acquisition across the thin sample; (b,c) example individual DPs of the (b) amorphous ring from the PS-rich phase; and (c) from the PEO-rich part. (d) Mean DP from one entire data set showing dominance of two rings, an inner ring near *d* = 4.6 Å and an outer ring near *d* = 3.8 Å; (e) BVM, summation of all the reflections that were detected in the data set using py4DSTEM; (f) virtual BF image from a 4D-STEM data set; (g–i) Example DPs from regions indicated on (f) by the corresponding colored boxes; (g) in purple are from an (*x*, *y*) position in real space with the amorphous ring and detector noise visible; (h) summed from 4 neighboring pixels; (i) summed from 16 neighboring pixels.

4D-STEM has been successfully applied to electron beam-sensitive materials such as organic polymers because the dose can be empirically minimized below the threshold for beam damage and because of its efficient use of diffracted electrons^16^ (Figure 2). The resulting structure maps can have spatial resolution of several to tens of nanometers, which is sufficient to probe the 40–60 nm domains in this block copolymer. 4D-STEM is a technique by which a convergent electron nanoprobe is rastered across a sample (two dimensions in real space), recording the diffraction pattern (DP) (two dimensions in reciprocal space) at each scan position.^17^ The DPs acquired from a 4D data set that can be used to reconstruct real space information about the phase distribution and orientation of the polymer material. In previous work, both phase map and orientation map have been used to show the crystal information inside soft-matter materials.^16,18−21^

One significant challenge of using TEM on polymer materials is the potential damage caused by the electron beam. However, methodologies exist to minimize and control beam damage, which include cryogenic imaging and low incident beam current.^22^ Additionally, direct electron cameras provide low noise and high speed for frame summing, which helps boost the signal above the noise floor of the camera. When operated in “counting mode”, noise incurred during camera readout can be suppressed resulting in even higher detector quantum efficiency. In our study, we implemented cryogenic conditions, utilized a direct electron camera in counting mode, and meticulously selected the 4D-STEM parameters, such as step size, aperture, and electron dose, to strike a balance between real space resolution and a reciprocal space signal-to-noise ratio (SNR).

Compared to other reported studies on organic materials,^16,19−21^ the PEO phase is very beam-sensitive and very weakly diffracting. Because there are no aromatic rings or chain conjugation, there are no π–π interactions, which often lead to long-range order that is detectable in a TEM camera. Instead, in the PEO phase, it is only the strongest reflections from the crystal structure that can be detected. The degree to which a polymer exhibits a long-range order is not only dependent on the material but also on the process; polyethylene for example, can exhibit either strong or weak diffraction.^18,20^ It was only through a combination of direct electron counting, cryogenic sample conditions, and optimal illumination conditions that diffraction was detected. In this paper, we have identified the experimental protocols and data analysis procedure to identify both the orientation of PEO crystals and the block copolymer morphology in a PS-*b*-PEO block copolymer using 4D-STEM. The diffraction data were transformed into polar coordinates, and the shape of amorphous domains was identified by an independent principal component analysis (PCA) approach. Juxtaposition of the outputs from these two procedures enables determination of the orientation of crystalline PEO domains relative to the PE-*b*-PEO lamellae.

## Experimental Section

### Materials

Polystyrene-*block*-poly(ethylene oxide) copolymers (PS-*b*-PEO) (200–222) were used, which were synthesized by anionic polymerization, as described in previous work.^23−26^ The molecular weight of the PS block was 200 kg/mol, and the molecular weight of the PEO block was 222 kg/mol. All electrolyte preparation was performed in argon-filled gloveboxes with less than 1 ppm of water and less than 1 ppm of oxygen to avoid contamination. PS-*b*-PEO was dissolved in (*N*-Methyl-2-pyrrolidone) NMP at 60 °C and stirred for 1 h. The mixture was free-cast onto a nickel foil on a casting plate to produce a flexible film. The film was dried under vacuum at 120 °C for 48 h.

### Preparation of Ultrathin Samples for TEM and 4D-STEM

Samples were sealed in a vacuum container for transfer. The vacuum container was opened in a glovebox, and the sample film is embedded in resin to protect the film sample. The sample was reinserted into a vacuum container and transferred out of the glovebox. A microtome EM UC6 (Leica Microsystems) was used to prepare the ultrathin sections. The sample was then transferred from the vacuum container to the microtome chamber. The cryo-microtome cuts the sample into 40–60 nm thick membranes (∼−120 °C), which were transferred cold to copper TEM grids featuring a lacey carbon supporting layer. Samples were sealed inside a sample container in a nitrogen atmosphere and then warmed to room temperature. The sample was then quickly transferred from a vacuum container into cryo holder (warm) and then transferred into the TEM. The procedure can be found in Figure S1.

### Staining with RuO_4_

As a comparison to the 4D-STEM images, some of the microtomed samples were stained with RuO_4_ for bright-field (BF) and high-angle annular dark-field (HAADF) imaging. These samples were placed over a staining jar and stained with RuO_4_ vapor for 10 min.

### TEM Bright Field

BF images from stained samples were obtained at an acceleration voltage of 120 kV using a JEOL-1200 equipped with a MegaScan camera (Gatan. Inc.). RuO_4_ preferentially stains the PEO rich region, and bright and dark regions can be attributed to amorphous and crystal-rich regions, respectively, in the BF-TEM images.

### STEM Image

HAADF images from stained samples were obtained at an acceleration voltage of 300 kV by using a FEI TitanX.

### 4D-STEM Acquisition

4D-STEM scans were acquired using the FEI TEAM I microscope at the Lawrence Berkeley National Laboratory using a Gatan K3 direct-electron-pixelated detector in counting mode. All scans were performed at −185 °C under liquid nitrogen cooling with a 300 kV accelerating voltage and a semiconvergence angle of ∼0.275 mrad, which yielded a diffraction-limited probe with a full-width half-max of ∼5 nm. The beam was rastered with a step size of 7–10 nm over one to several micrometers field of view. The electron dose per sample area over the entire scan is ∼90 e^–^/Å^2^. Gold nanoparticles were used to calibrate the reciprocal space pixel size as well as measure the elliptical distortion present in the data set.

### Data Analysis Method

The code used for this work is primarily based on open source py4DSTEM repository (https://github.com/py4dstem/py4DSTEM).

## Results and Discussion

### Morphology of PS-*b*-PEO

This work focuses on a high molecular weight PS-*b*-PEO with a lamellae morphology, and the widths of the PEO lamellae ranged in the micrographs from 40 to 60 nm (Figure 1d–f). Some of the variation in widths is due to the fact that some of the lamellar normals are not in the plane of the specimen. To confirm the lamellar structure, PS-*b*-PEO samples were stained with RuO_4_, and the morphology was examined in TEM. A HAADF image obtained in STEM mode at 300 kV is shown in Figure 1d, in which the bright part is the PEO-rich region. The BF images obtained in TEM mode at 80 kV are displayed in Figure 1e,f, where the dark portions correspond to the PEO-rich areas, and the bright portions represent the PS-rich regions. In the sample regions selected, the electron beam direction is mostly perpendicular to the sample piece and parallel to the PEO–PS interfaces; the sample is thin enough that in most regions of the sample, blocks extend through the thickness of the sample. There are some regions where two block orientations overlap, and this can be seen for example in the lower right of Figure 1d.

### 4D-STEM Approach

A schematic of the 4D-STEM experiment is shown in Figure 2a. There are two length scales of concern. The first is the reciprocal space resolution, the pixel size of the diffraction detector. A polymer as weakly diffracting as the PEO phase requires a small probe convergence angle to concentrate the electrons to a small number of pixels. Even so, the data were binned by four in postprocessing to increase the SNR to result in a pixel size of 0.0021 nm^–1^, which translates to a *d*-spacing precision of ±0.002 nm. This defined the limit to which reflections with similar *d*-spacings could be separated. The second length scale of concern is the pixel size of the real-space structure map, which is determined by the step size between probe positions. The probe convergence used here resulted in a probe fwhm of 5.3 nm; it is known that the tails of the Airy disk profile extend beyond this dimension and that secondary electrons scatter in the lateral direction causing the loss of the long-range order even beyond the tails.^27^ As such, it was determined empirically that a 7–10 nm step was the smallest that the sample could sustain with this probe diameter and provided interpretable diffraction data. Consequently, each 40–60 nm lamella contains four to six pixels across its width.

The particular data set featured in Figure 2 used a 10 nm step with 200 × 200 scanned positions resulting in a structure map of 2 μm × 2 μm and 4 × 10^4^ DPs. The raw data were binned in reciprocal space by 4, such that each pattern is approximately 512 × 512 pixels. The pixel width in reciprocal space is 0.0021 Å^–1^. Normal centering of the DPs to account for beam sway during scanning was performed. Figure 2d shows the mean DP from the entire data set; two strong rings from the PEO phase are evident. These can be identified as diffraction corresponding to *d* = 4.6 Å (inner ring) and *d* = 3.8 Å (outer ring). A Bragg vector map (BVM) is shown in Figure 2e, which shows all of the possible reflections that were identified by the cross-correlation with the library. Rings at 4.6, 3.8, and 3.4 Å are evident as well as of higher order.

Representative DPs are shown in Figure 2b,c,g, and a virtual BF image constructed from the data set is shown in 2f. Virtual images apply virtual apertures or combinations of apertures at 4D-STEM postprocessing to explore distribution of diffraction signals in real space.^18,22,28^Figure 2b shows a typical DP from the amorphous phase, and Figure 2c shows that from the PEO crystal phase. Those in purple are from 1 probe position, those in yellow arise from 4 neighboring pixels summed (real-space binning ×2, Figure 2h), and those in green are from 16 neighboring pixels (Figure 2i). Of note is the fact that the single frame patterns (purple) are weak, noisy, and sparse; only 1–3 Friedel pairs of reflections are present in each frame, and in most cases, only 1 pair is detectable. While summing frames improves the SNR in the pattern, the real space discrimination is lost. It is evident that the amorphous ring of the PS and also of the amorphous regions of the PEO lie on top of the PEO reflections, increasing the background level. In order to keep the 10 nm pixel size in the structure map, it was necessary to process the individual frames without binning them in real space. Typically, with a 4D-STEM data set, the single frame DP is cross-correlated to the library of patterns that are possible for the known crystal structure and the Bragg reflections are identified. This method was attempted but produced some false positives due to the strong signal from the amorphous phases in some of the frames.

### Electron Diffraction from PEO

The PS-*b*-PEO samples exhibit a weak long-range order, and only the first-order reflections are detectable. Table 1 lists the strongest reflections, the *d*-spacing values, and their predicted intensity generated from the .cif file of pure PEO. We examined the strongest possible reflections corresponding to a *d*-spacing of ∼3.8 Å (032), (−132), (112), and (−2 1 2) and the (120) reflections at 4.6 Å and some weak signal at 3.4 Å (024) and (2 2–4).

**Table 1:** Simulated Diffraction Peaks, Corresponding *d*-Spacing, and Intensity of Pure PEO

reflections	*I*/*I*_max_ (%)	*d*-spacing (Å)
(1 2 0), (1̅ 2̅ 0), (1̅ 2 0), (1 2̅ 0)	34.0	4.625
(1 1 2), (1̅ 1̅ 2̅), (1̅ 1 2̅), (1 1̅ 2)	53.4	3.857
(0 3 2), (0 3̅ 2̅), (0 3̅ 2̅), (0 3 2̅)	100	3.813
(1 3 2̅), (1̅ 3̅ 2), (1̅ 3 2), (1 3̅ 2̅)	74.8	3.786
(2̅ 1̅ 2), (2 1 2̅), (2̅ 1 2), (2 1̅ 2̅)	54.0	3.775
(0 2 4), (0 2̅ 4̅), (0 2 4̅), (0 2 4̅)	30.2	3.391
(2̅ 2̅ 4), (2 2 4̅), (2̅ 2 4), (2 2̅ 4̅)	36.3	3.316

**Figure 3:**
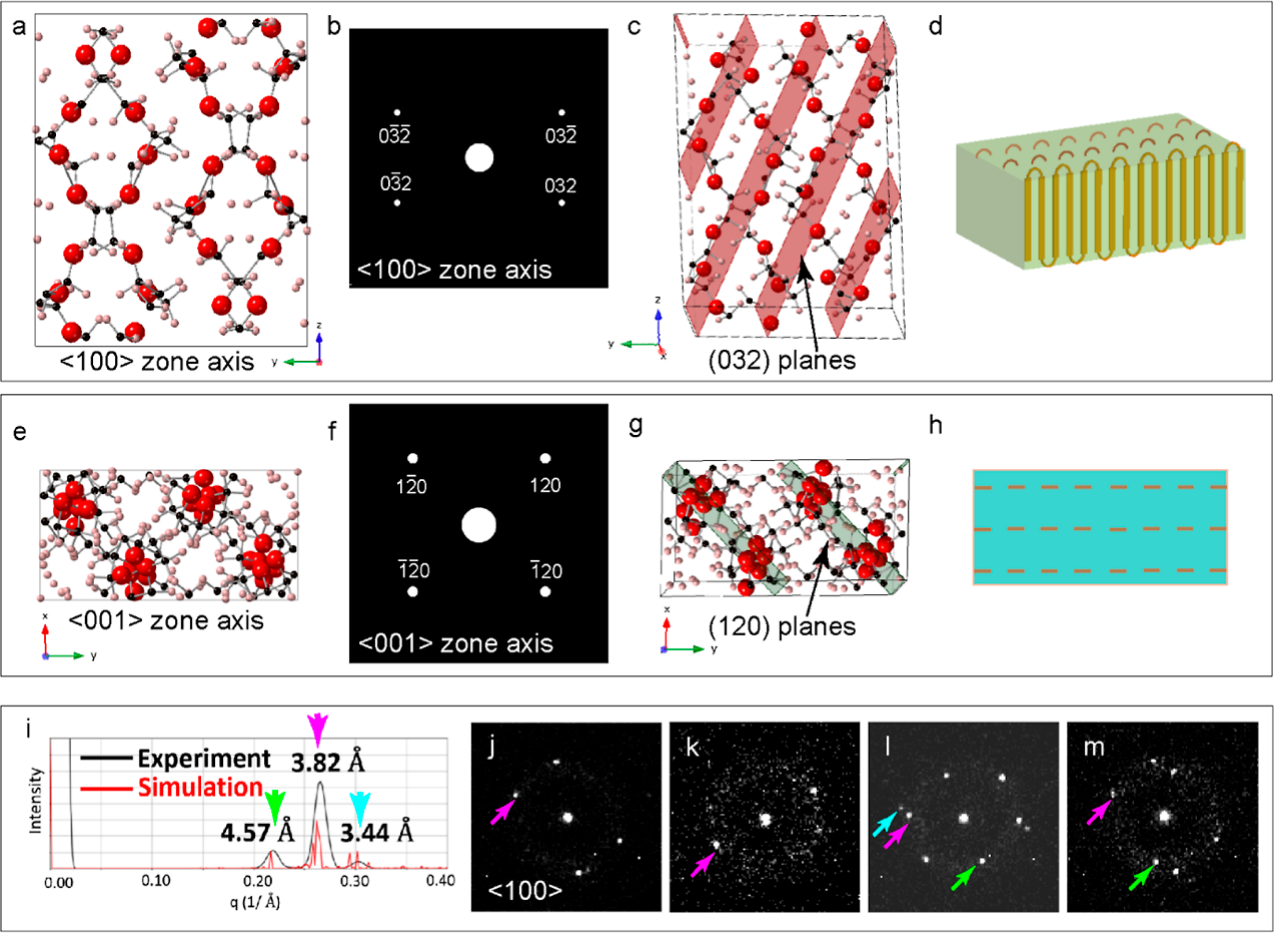
Electron diffraction from perhaps monoclinic PEO crystals; (a) *x*-axis projection from .cif file; (b) simulated diffraction showing only strongest reflections of {032} at *d* = 3.8 Å along the <100> zone axis; (c) (032) planes shown in red; (d) cartoons of PEO domains; (e) *z*-axis projection; (f) simulated diffraction showing strongest reflections of {120} at *d* = 3.4 Å; (g) (120) planes in green; (h) cartoons of PEO domains in <001> zone axes; (i) power spectrum simulated using py4DSTEM in red and generated from the BVM of the data set shown in black; the dominant reflections are labeled. This work does not have the reciprocal space resolution to separate the multiple rings contributing to each of the three-*q* indicated with green (*d* = 4.6 Å), magenta (*d* = 3.8 Å), and turquoise (*d* = 3.4 Å). (k–n) Example DPs; (j) four reflections with *d* = 3.8 Å, consistent with simulated diffraction along the <100> zone axis shown in (b); (k) only two reflections with *d* = 3.8 Å are often observed; (l,m) patterns show reflections corresponding to both <100> and <001> zone axes due to the presence of multiple crystals in the scattering volume or the effect of binning.

A projection down the ***x*** axis of the unit cell (<100> zone axis), its simulated DP, and the planes generating the (032) signal are shown in Figure 3a–c. Figure 3e–g shows similar relationships for the <001> zone axis down the ***z*** axis of the unit cell.

From the .cif file, a simulated electron diffraction powder spectrum was generated using py4DSTEM-ACOM (Automated Crystal Orientation Mapping),^29^ and this is shown as the red curve in Figure 3i. The black curve is a histogram of a BVM generated from experimental data using ACOM, which contains all of the diffraction peaks detected. As previously mentioned, there may be false positives, but the curve here shows good agreement. It is instructive to note that because of the need to heavily bin in reciprocal space, the data lacked the reciprocal space resolution to separate the four dominant peaks at ∼3.8 Å. Figure 3j–m shows example DPs summed from 4 neighboring scan positions amounting to an area of 20 nm × 20 nm. Reflections at a given *d*-spacing or *q* value are indicated with a colored arrow according to the colors in Figure 3i. Figure 3j shows one domain that is on the <100> zone axis as it matches the pattern. Figure 3k exhibits one Friedel pair and a fair amount of amorphous ring–probably one domain off of a symmetric zone axis. Figure 3 shows multiple reflections at different *q* values, which are most likely from more than one domain. Multiple reflections can either be because the probe is sampling adjacent domains, or it can be that there are domains on top of one another through the TEM thickness layer. The microtome layer was estimated to be 40–60 nm in thickness, and since the lateral domain size is measured at 10–30 nm, it is expected that the probe may sample more than one domain through the thickness. Reflections in the DP will only be present if the domain is satisfying the Bragg condition, so domains can exist that produce no measurable signal. A cartoon of a domain is shown in Figure 3d,h. As is common for polymers, the undulating polymer chains are shown to predominantly follow the **z**-axis of the unit cell. While the unit cell has only two 90° angles and the third is 125.4°, it is not known if the crystallites or domains follow the unit cell shape; they are schematically represented in this article as a cuboid to simplify the representation, but one domain is actually composed of a number of unit cells.

### PS-*b*-PEO Phase Map

Conventionally, a virtual dark-field image could be created from the signal in a specified annular range, but because the PS amorphous scattering is in the same annular area as reflections from the crystalline PEO, this is not the best way to generate a structural map that will show contrast between the PS and PEO phases. Instead, a virtual phase map was generated by processing the median from the polar coordinates (Figure S2a–h) through PCA.^30,31^

**Figure 4:**
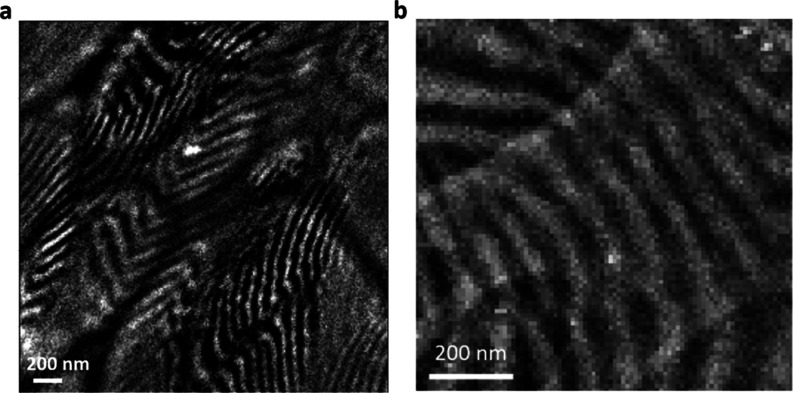
Virtual dark-field images were generated from the PCA method, one with a large field of view (a) and one with high resolution (b). The bright areas represent the PS-rich portions, and the dark areas represent the PEO-rich regions.

This phase map from the PCA provided a clear visualization of the lamellae structure, where the bright areas represent the PS-rich portions and the dark areas represent the PEO-rich regions. Comparing the phase map (Figure 4b) with the image where contrast was obtained from the RuO_4_-staining PS-*b*-PEO sample (Figure 1f), the features are similar. One significant advantage of using 4D-STEM instead of staining is that no toxic chemicals are required to obtain image contrast. Additionally, unlike in heavy element staining experiments, the sample has not been altered prior to imaging. Additionally, the lamellae spacing, which is approximately 40–60 nm (4–6 pixels for a 10 nm step size), can be accurately measured from the virtual DF image, and the value is consistent with the RuO_4_-stained HAADF images. Furthermore, when using the analysis of the 4D-STEM data, we can generate a virtual detector in the DPs to show its real space distribution such as the domain size statistics as described below. The 4D-STEM approach can provide detailed information not available from traditional RuO_4_ staining methods.

### Orientation Map and Domain Size Statistics

**Figure 5:**
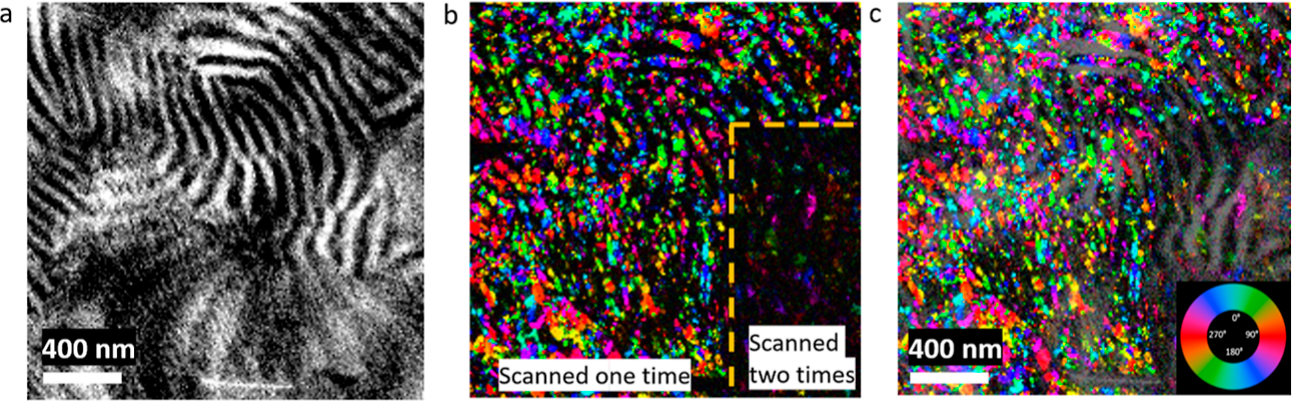
Structure maps of PS-*b*-PEO: (a) phase map is constructed by using the second principal component from a PCA analysis to weight the intensity in which the dark region is the PEO-rich phase and the bright region is the PS-rich part; (b) orientation map of the PS-*b*-PEO from the strongest reflection in the *q* value range corresponding to the 3.8 Å *d*-spacing, the color is related to the orientation of the domain inside the PEO-rich part [black region in figure (a)]; beam damage is shown in the lower right, where the signal is diminished because of a second illumination in that region; and (c) combination orientation map with both figure (a,b).

Figure 5 shows an orientation map constructed from the strongest pair of reflections at each probe position for the *q* value range corresponding to the *d*-spacing of 3.8 Å. The reflections were identified using the polar method of peak ID described above and are shown in Figure S2f–h. In the Figure S2h step, once the median was defined for each bin, the reflection was identified above the median and the coordinates recorded. The second and third strongest pairs can also be constructed; their phase maps are shown in Figure S3. One problem is that it is challenging to visualize domains that are on top of one another with these orientation maps. Often times, grains that are stacked are visualized with lines, so that one can see multiple layers. However, as is evident from Figure S3, the number of scan positions that contained frames with more than one Friedel pair is small, as evident from the few pixels with color and the large number of black pixels. In Figure 5b, electron beam damage can be visualized. In the lower right quadrant of the image, the signal clearly decreased after scanning the same region two times. This is direct visualization of electron beam damage, which showed that the PS-*b*-PEO samples are very electron beam-sensitive.

**Figure 6:**
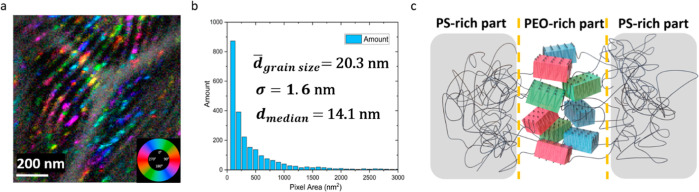
Domain size statistical results: orientation map constructed from the strongest reflections and domain size; pixels of same color have their strongest pair of reflections oriented in the same direction; (a) high magnification orientation map using 7 nm step size; (b) mean domain size distribution from four data sets that contained 2000, mean domain size is 20 nm with standard deviation between data sets = 1.6 nm, and the median domain size is 14 nm; and (c) 3D model built based on the statistical results (illustrated size).

The orientation map provides important insights into the orientation of small domains within the PEO-rich part of the PS-*b*-PEO sample. The color represents different orientation angles corresponding to the legend shown in Figure 5c. It is clear that inside the PEO regions, these domains exhibit a range of in-plane orientations. In Figures 5b and 6a, orientation maps from a large region (2500 × 2500 nm, step size 10 nm) and a small region (1050 × 1050 nm, step size 7 nm) are presented. In Figure 6a, with a 7 nm step size, the distribution of orientation domains can be directly seen inside the PEO-rich part. The in-plane orientations that are continuous provide a clue about the domain size. In Figure 6b, the number of pixels of a given domain orientation that are continuous was counted.

This method of image segmentation is sometimes called “seeded region-growing”^20,32^ in which the nearest neighbors are queried to see if they meet a set of criteria. If one of them does, it is added to the original seed, and the pixels adjacent to the new pixel are queried. In this method, there are several defined criteria. First, the “same orientation” is defined as being within 5° allowing for some bending and twisting of the domains. Second, the number of domains through the thickness of the TEM sample was limited to 4 (in fact, the thickness of the TEM sample is such that it is more likely that only 2 or 3 domains are present through the thickness, 4 is unlikely). Lastly, there were two threshold values of intensity: one to define a “seed” and a less stringent threshold to “grow” the seed. This threshold is analogous to the median used in the construction of the phase map, as shown in Figure S2h. Results from this analysis of 4 data sets representing 2 × 10^3^ domains generated a histogram of domain sizes in terms of the area shown in Figure 6b. According to the histogram, the average area of a domain inside the PEO block is 410 nm^2^. This area converts to a domain with a cube length of 20 nm; the standard deviation between data sets is 1.6 nm; the median domain size is 14 nm. It is obvious that most domains are limited to 10–30 nm. The schematic in Figure 6c shows the size of the domain compared to the lamellar spacing (the orientation here is for reference only and will be discussed in the next section). One thing worth mentioning is that in the orientation map, most domains are elongated in one direction; the domain size reported in this work is calculated from the area, which was measured in the data. In fact, there is likely a long domain direction and a short domain direction.

### Relative Angle and Proposed Model

Ultimately, we would like to understand whether there is a preferred orientation of the PEO crystallites with respect to the larger length scale of the PS–PEO interface. Ion transport can only move lengthwise through the PEO amorphous phase; it does not cross the PS–PEO interface and transport in the PS phase.^33^ One could imagine that the PS–PEO interface might, through either steric constraints or charge/bonding at the interface, cause the PEO to order in some fashion. Since the data from the PS-phase map exist, it is possible to define a director of the pixel that is perpendicular to the closest PS–PEO interface. The orientation of a specific plane reflection can now be correlated to see if there is a preference for alignment.^30^

**Figure 7:**
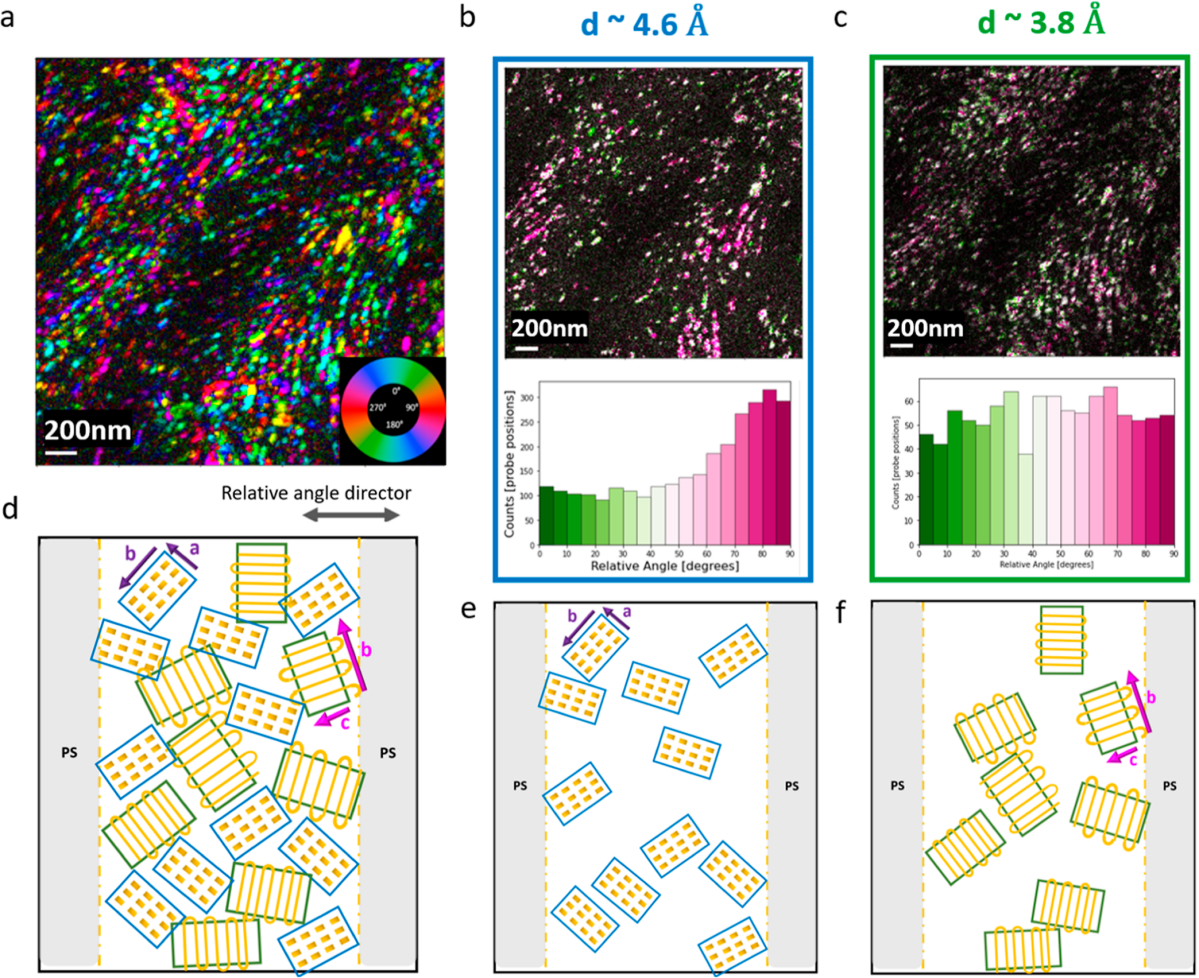
Relative angle and proposed model based on relative angle results: (a) orientation map of the strongest reflection in the *q* range corresponding to the 3.8 Å *d*-spacing with step size 10 nm, 2500 nm × 2500 nm region; (b,c) top: map of angle between strongest reflection in *q* and the perpendicular direction of the PS–PEO interface, the green means 0° with the PS–PEO interface, the pink means 90° with the PS–PEO interface; bottom: histogram of the angle; (b) reflections corresponding to *d*-spacing of 4.6 Å, which is assigned to the {120} family of reflections; (c) reflections corresponding to *d*-spacing of 3.8 Å; (d) schematic top view inside lamellae PEO part: the blue domain is represented by *d*-spacing 4.6 Å and the green domain is represented by 3.8 Å; (e) top view inside the PEO-rich part with all *d*-spacing 4.6 Å domains; (f) top view inside the PEO-rich part with all *d*-spacing 3.8 Å domains.

The innermost diffraction ring at a *q* value of 4.6 Å can be assigned to the (120) reflections as there are no other possible reflections of high intensity within this *d* spacing. Indeed, some of the frames show the square pattern of the 120 reflections of the <001> zone. An orientation map was generated from this *q* and is shown in Figure 7b. The histogram of angles of this *q* with respect to the director of the PS map indicates a moderate preference for the (120) planes to be parallel to the PS–PEO interface. This preference for the (120) planes to be parallel to the interface can be quantified as about 2.3 ×, as 280 domains are parallel to the interface, while 120 domains are perpendicular, and the incidence is approximately monotonic across the director angle. A second data set was analyzed; the results are shown in Figure S4 and the preference was found to be 1.6×. Neither data set shows a large preference, but one wonders if these domains are closer to the interface and affected by the steric constraints of bonds or charge at the interface. Due to the prevalence of beam damage, we were unable to acquire with a step size less than 7 nm and consequently do not have the spatial resolution to distinguish between domains in the center of the block and those closest to the interface, but this correlation would be interesting to study in the future.

An orientation map is constructed from the strongest reflection in the *q* value corresponding to *d* = 3.8 Å and is shown in Figure 7c. Again, a correlation of the second ring reflections to the director of the interface is made, and this histogram is shown in Figure 7c. Figure 7c shows that all of the orientations are probably similar. It is important to note that unlike Figure 7b, which represents one family of planes, Figure 7c contains reflections from possibly four different families of planes. While their collective behavior does not show an angular preference, it is possible that with better reciprocal space resolution, they could be separated.

We find that domains that are aligned such that the (120) reflections (*d*-spacing ∼4.6 Å) are in the Bragg-scattering condition have a slight preference for ordering the (120) planes parallel to the PS–PEO interface. Domains that align the <001> crystal axis with the electron beam are an example of all four {120} reflections being excited, but there are other off-zone conditions where only two of the {120} reflections are visible in the DP. In the specific case of the <001> orientation, the *c*-axis is parallel to the PS–PEO interface. The most common diffraction signal from the sample arises from a *d*-spacing near 3.8 Å. The reciprocal resolution of the data does not allow for separating between the reflections from the four groups of planes corresponding to the (032), (−132), (112), and (−2 1 2) reflections, so we treat these four “flavors” as a collective. As a collective, the domains that generate this 3.8 Å signal do not appear to be aligned in any specific direction with respect to the PS–PEO interface. We cannot determine if one or more of these flavors is preferentially oriented, but the collective, as a whole, is not. To our knowledge, this is the first report that measures an orientation of individual domains, as opposed to spatially averaged, with respect to the morphology of the block interface.

### New Model based on 4D-STEM Results

Based on the 4D-STEM results presented in this study, we propose a new model for the structure of PS-*b*-PEO. Our model is based on a comprehensive analysis of the DPs and virtual images obtained from 4D-STEM, which provides new insights into the morphology and orientation of the lamellae. The structure of PS-*b*-PEO can be regarded as three levels of order at different length scales. The order with the smallest length scale is the polymer chain and unit cell and formation of individual crystalline domains. The second order is the orientation of the domains and disordered PEO chains inside the PEO-rich phase. The third order is the block copolymer morphology of both the PEO and PS phases. In the PS-rich phase of the sample, the crystal structure is entirely amorphous, as made evident by the DPs that show only an amorphous ring. However, inside the PEO-rich part, we observed small domains with a size of 10–30 nm, with the mean domain size being 20 nm with standard deviation of 1.6 nm; the median domain size is 14 nm. For many of these domains, there does not appear to be a preferred orientation with respect to the PS–PEO block interface. For some of the grains that exhibit {120} diffraction, there appears to be a slight preference for domains to align with the (120) planes normal to the PS–PEO interface. Importantly, our model provides new second order information about domain behavior that cannot be obtained by using traditional TEM methods. Specifically, the 4D-STEM results allow us to directly observe and quantify the domain size and orientation within the PEO-rich part of the sample.

Kanomi et al. studied the chain tilting within the polyethylene (PE) lamellae using the low-dose 4D-STEM method.^21^ This study, for the first time, provides nanometer-scale resolution in positional space and reveals direct evidence for the existence of different inner-chain orientations within lamellar crystals. In contrast to the neat PE thin film comprising well-ordered large lamellae, which is up to 500 nm long and a few tens of nanometers thick, the PEO block in the PS-*b*-PEO diblock copolymer exhibits the presence of crystallites with much smaller dimensions dispersed in the amorphous regions within the microphase separated domains. This poses a significant challenge when attempting to image and analyze the crystallinity and orientations due to these crystallites’ smaller size and dispersion. Furthermore, the presence of interfaces between the PS and PEO components also complicates an accurate analysis. To address these challenges, we optimized the data collection conditions and employed the py4DSTEM software package, which can generate a larger field-of-view orientation map, quantitively measure domain sizes, and calculate the relative angle of domains.

We believe that our results are critical for understanding the mechanical and physical properties of these materials, particularly their electrochemical performance. Our proposed model is consistent with previous models based on average X-ray scattering data, but it provides a more detailed and comprehensive picture of the lamellar structure of PS-*b*-PEO based on direct observations and measurements with a spatial resolution of ∼10 nm.

## Conclusions

In this work, we investigated the structure within the PEO-rich component of the lamellae morphology of PS-*b*-PEO, a promising material for use as a polymer solid-state electrolyte. Previous studies based on X-ray diffraction suggested that the structure within the PEO domain was polycrystalline and randomly oriented. However, no experimental evidence of this model existed. Using cryo 4D-STEM, we successfully generated orientation maps of PS-*b*-PEO that suggest that at least some of the crystallites exhibit a preferential alignment with respect to the PS–PEO interface. Our findings represent the first time that researchers have directly measured a specific domain orientation with respect to the block interface within the lamellae structure of a semicrystalline block copolymer.

We believe that the techniques and methodologies developed in our study can be applied to other electron beam-sensitive semicrystalline materials. Our research serves as a foundation for future studies that aim to directly observe the crystalline domain orientation inside complicated semicrystalline block copolymers, ultimately providing a deeper understanding of their structure and properties.

## Supporting Information Available

PS-*b*-PEO 4D-STEM sample preparation procedure; polar method of peak ID; PCA method; phase map from different *d*-spacing signals; and orientation map of the PS-*b*-PEO in different areas (PDF)
Crystal information (CIF)

## Supplementary Material

ma3c02231_si_001.pdf

ma3c02231_si_002.cif
