# Supplementary material for: Direct Imaging of the Crystalline Domains and Their Orientation in the PS-b-PEO Block Copolymer with 4D-STEM
Source: Macromolecules. 2024 Apr 18;57(12):5629–38. doi: 10.1021/acs.macromol.3c02231 (PMC11210284; doi:10.1021/acs.macromol.3c02231)
Supplement: Supplementary file 1 — ma3c02231_si_001.pdf [file ma3c02231_si_001.pdf]

Supporting Information for

Direct imaging of the crystalline domains and their orientation in the PS-b-PEO block copolymer with 4D-STEM

Min Chen[1,2,3], Karen C. Bustillo[3], Vivaan Patel[2,4], Benjamin H. Savitzky[3], Hadas Sternlicht[1,3], Jacqueline A. Maslyn[2,4], Whitney S. Loo[2,4], Jim Ciston[3], Colin Ophus[3], Xi Jiang[2], Nitash P. Balsara[2,4], Andrew M. Minor[1,2,3]*

[1]Department of Materials Science and Engineering, University of California, Berkeley, CA, 94720 USA.

[2]Materials Science Division, Lawrence Berkeley National Laboratory, Berkeley, CA, 94720 USA.

[3]National Center for Electron Microscopy, Molecular Foundry, Lawrence Berkeley National Laboratory, Berkeley, CA, 94720 USA.

[4]Department of Chemical and Biomolecular Engineering, University of California, Berkeley, CA, 94720 USA

*Corresponding author: Aminor@lbl.gov

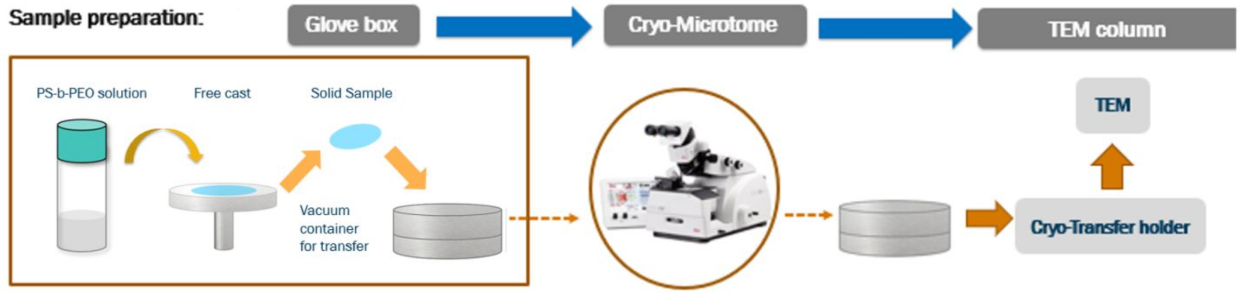

Figure S1. Sample preparation procedure: process was designed to avoid air exposure; (1) PS-b-PEO solution was first free cast in glove box and then sealed in vacuum container for transfer (2) Vacuum container opened in glove box and sample film is embedded in resin and which protects the film sample, sample was reinserted into vacuum container and transferred out of glovebox (3) Transferred vacuum container to the microtome chamber (4) Cryo-microtome cut the sample into 40-60nm thick membrane and transferred cold to TEM grid (5) Sample was sealed inside sample container in nitrogen atmosphere and then warmed to room temperature. (5) Sample was quickly transferred from vacuum container into cryo holder (warm) and into TEM. (6) Sample was imaged at cryo conditions. This short exposure during the transfer from the vacuum container to the TEM only works for the HMW SEO. Lower molecular weight samples will have to be cryo-transferred into the TEM to prevent air exposure.

Polar method of Peak ID

The diffracted signal from the PEO phase was, by nature, weak and highly beam-sensitive. In addition, the amorphous ring of the polystyrene phase was coincident in q and thus modulations in the scattering of the polystyrene phase affected identification of the PEO spots. Typically, a .cif file can be loaded into simulation to identify the myriad of possible electron diffraction patterns and those simulated diffraction patterns can be cross-correlated to the experimental data and a zone axis and orientation defined. In the case of the PS-b-PEO, due to weak long-range order and presence of twists and bends within the PEO phase, many of the diffraction patterns acquired were not "on-zone" and therefore exhibit only the first-order reflections and often only one or two Friedel pairs and not the full pattern. These partial patterns, combined with the aforementioned coincidence of the polystyrene and poly-ethylene amorphous ring, made identification of the exact pattern ineffective. Considering this, the whole diffraction pattern fitting method was not optimal, and a new method was needed. A new methodology using polar coordinates was therefore used to create a meaningful representation of the data where diffraction spots could be identified above a threshold set by the amorphous scattering ring at the same q value. So, instead of performing a whole pattern fitting to each DP, the data was transformed into polar coordinates. SI Figure 2a shows the mean diffraction pattern and the transformation to polar coordinates in SI Figure 2b. In this transformation, the data is "folded over" to account for the 2-fold symmetry of the reflections. SI Figure 2c and 2d shows one frame and its polar transform respectively. Still, in SI Figure 2d,

one can see that there is amorphous signal at the same q value as the reflections and so the detection of the reflection relies on defining a threshold value, below which, the signal will be ignored. The advantage to using polar coordinates within a well-defined range of q value or radius, is that in the angular direction, the noise level is constant, and a median can be a good representation of the threshold. Virtual detectors in the shape of an annulus with a width of 2 pixels were defined from the center of the diffraction; this resulted in virtual detectors of concentric rings or "segments" (SI Figure 2f). 80 segments were defined from the center to the outer edges covering 160 of the 256 pixels. The signal from each concentric ring was transformed into polar coordinates after folding the Friedel pairs onto each other (SI Figure 2g). The resulting image was now a strip of data where θ is on the long axis from 0° to 180°, and it had a width of 2 pixels. A Gaussian blur was applied to the data to make the noise more monotonic. If the particular bin corresponding to a q value (or radius of diffraction pattern) contained a crystalline reflection, there would be a spot. Plotting the polar data as a function of theta for a bin near the 3.8 Å reflections would look like SI Figure 2h.

Principal Component Analysis (PCA) method

We chose the median signal as the magnitude for the amorphous component. This has the effect of thresholding the data so that we removed the high intensity reflections and just addressed the background signal. Now each reconstructed real space image (200×200 pixels) contained a value of the median in the real space pixel and there were 80 of them in a stack corresponding to each 2-pixel bin in q value. The signal from the amorphous PS phase was distributed among the q but it was not known a priori where or at what magnitude. We performed principal component analysis on this stack of images. Each component is shown in SI Figure 2i. The first component is most likely the thickness. The second principal component (SI Figure 2j) when used to weight the

segments, produced an image that looked highly correlated to the HAADF image showing the PS and PEO lamellar blocks as shown in SI Figure 2l-m. We took this to be the signal from the PS, and subsequently generated a phase map of the PS-PEO from this second principal component (SI Figure 2l-m).

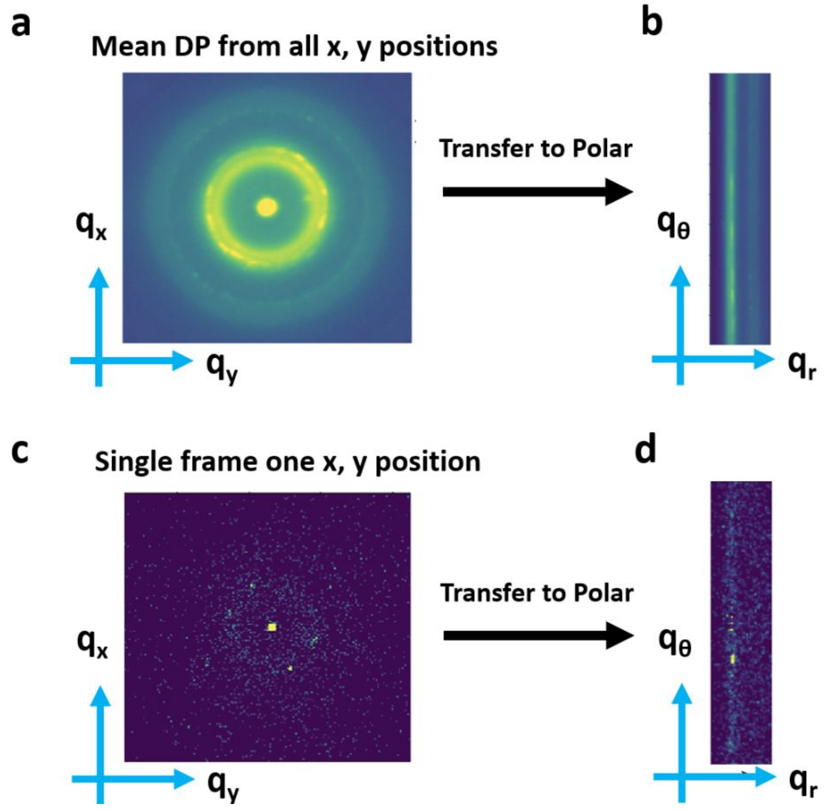

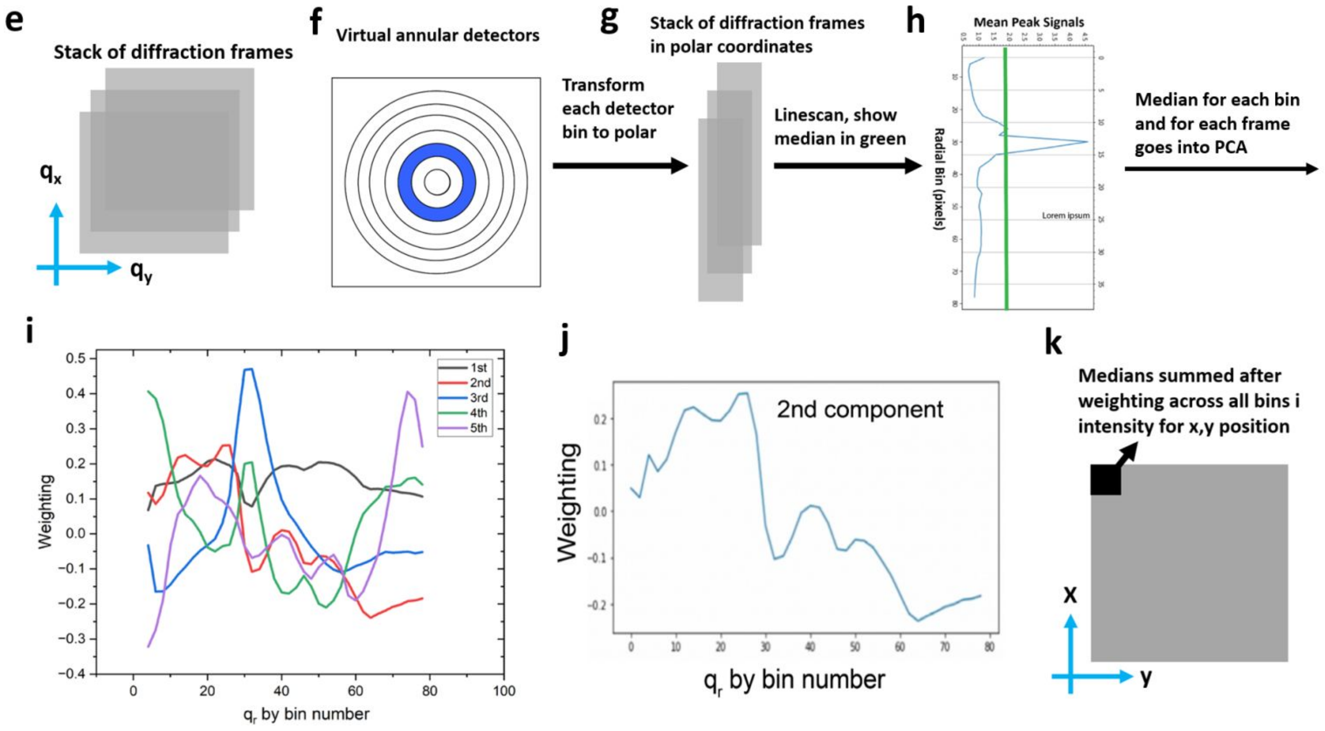

Figure S2. Polar method of peak identification. a) Mean diffraction from all x, y scan positions; b) Frame in a has been folded over to sum the 2-fold symmetry and then converted to polar coordinates, the y-axis is theta from 0 to 180 degrees, the x-axis is the radius; c) Similar to a) but for one frame only; d) Similar to b) but for one frame; e) Schematic reminder that each x, y position is one frame; f) Each pattern was segmented into annular regions with a two-pixel widths. Each region is called a radial bin for the purposes of the principle component analysis (PCA); g) Schematic reminder that each x,y frame is transformed into a stack of polar images that are two pixels wide; h) Intensity of one of the polar images with green line marking the median intensity, median intensity values from each bin and each x, y position were fed into PCA; i) Different components of the PCA produced weightings for each radial bin; j) second component of the PCA produced weighting; k) Structure map is constructed by using second component, because it matches the PS-PEO phase map, we assign the second component to the PS amorphous ring.

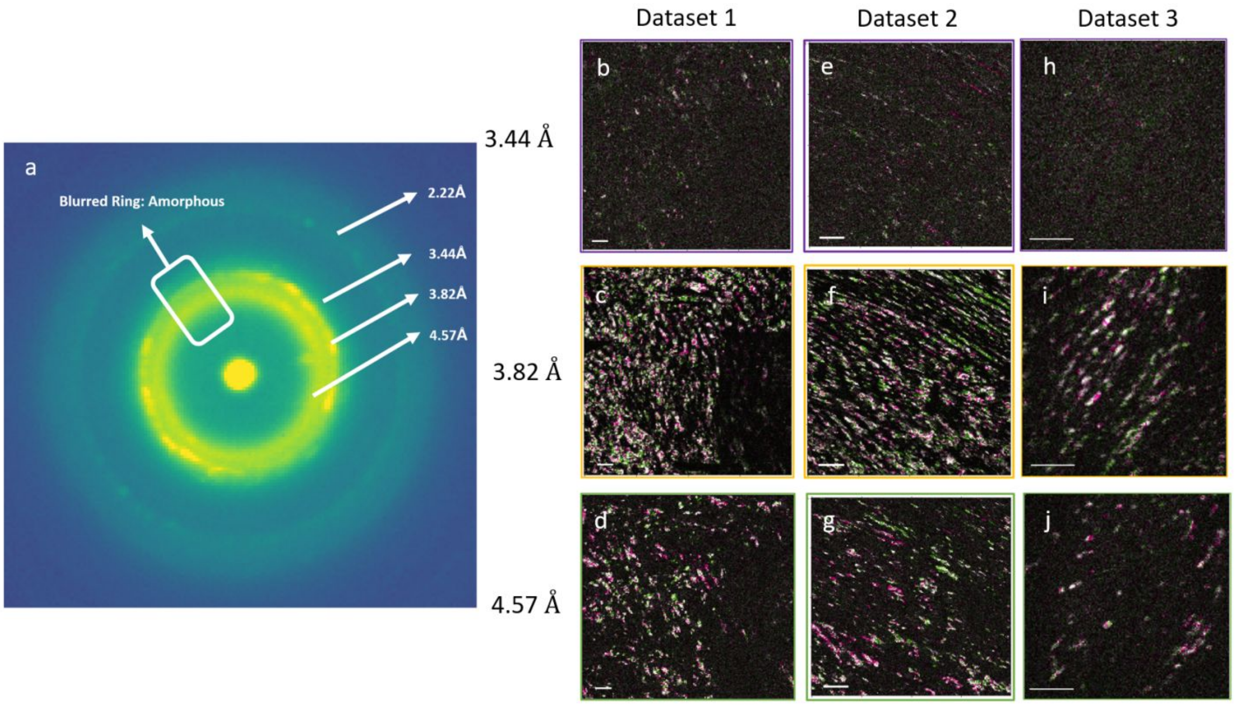

Figure S3. a) Mean diffraction pattern; phase map from different d-spacing signals, the scale bars are all 200nm, the green means 0° with the PS-PEO interface, the pink means 90° with the PS-PEO interface: b-d) with step size 10 nm; e-g) with step size 8 nm; h-j) with step size 7 nm.

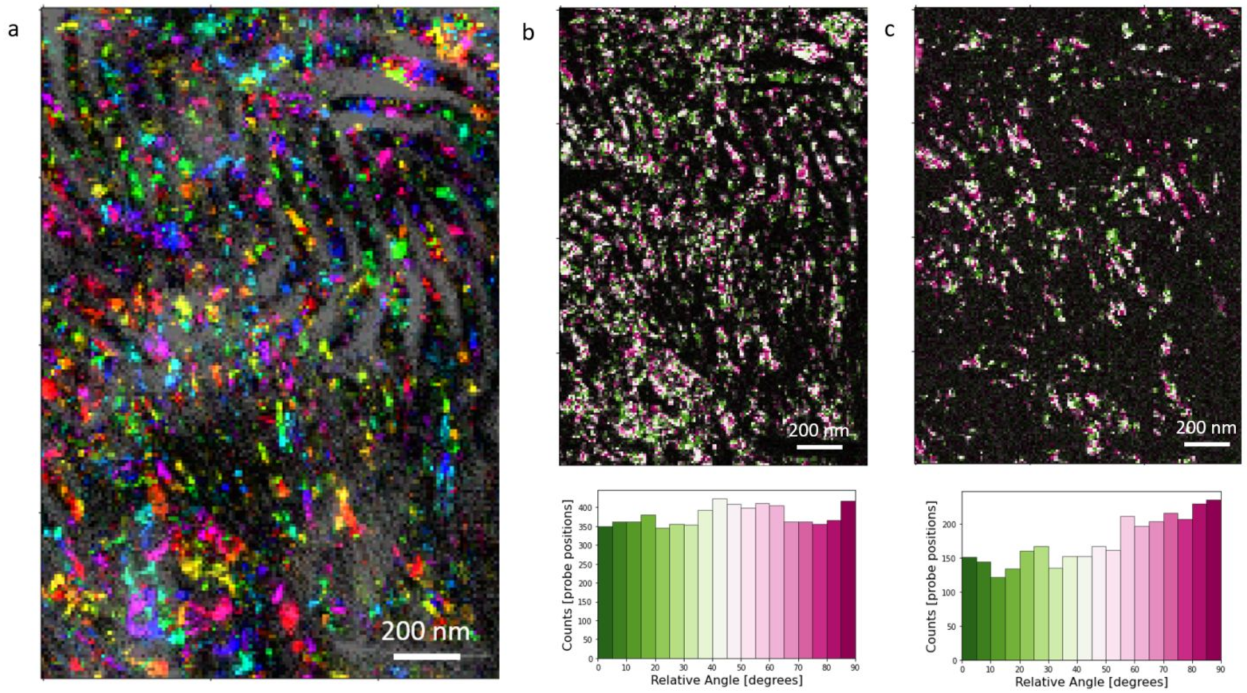

Figure S4. a) Orientation map of the PS-b-PEO, the color is related to the orientation of the domain inside the PEO-rich part, same as figure 5c to better compare the relative angle b) Relative angle and proposed model based on relative angle results, Zone axis [100] pixel relative angle with PS-PEO interface map, the green means 0° with the PS-PEO interface, the pink means 90° with the PS-PEO interface. Histogram below: orientation distribution of all pixels; c) Zone axis [001] pixel relative angle with PS-PEO interface.
